# Prioritization Patterns of Nurses in the Management of a Patient With Delirium: Results of a Q‐Methodology Study

**DOI:** 10.1002/nur.22449

**Published:** 2025-02-02

**Authors:** Luisa Sist, Matteo Pezzolati, Nikita Valentina Ugenti, Silvia Cedioli, Rossella Messina, Stefania Chiappinotto, Paola Rucci, Alvisa Palese

**Affiliations:** ^1^ Department of Biomedical and Neuromotor Sciences Alma Mater Studiorum University of Bologna Bologna Italy; ^2^ Sviluppo Professionale e Implementazione della Ricerca nelle Professioni Sanitarie (SPIR), IRCCS Azienda Ospedaliero‐Universitaria di Bologna Bologna Italy; ^3^ Ausl Romagna Italy; ^4^ Department of Medicine University of Udine Udine Italy

**Keywords:** delirium, delirium/nursing, management, prioritization, priority setting, Q‐methodology

## Abstract

Nurses are required to decide on priorities; however, how they prioritize the interventions toward patients with delirium is still unclear. Therefore, expanding the knowledge on (a) how nurses prioritize interventions to manage episodes of delirium and (b) the underlying prioritization patterns were the aims of this study. The Q‐methodology was applied in 2021. A systematic review to identify the recommended interventions for patients with delirium was performed, and a nominal group technique was used to select those interventions that are applicable in daily practice (35 out of 96 identified). Then, using a specific scenario, 56 clinical nurses working in hospital medical (*n* = 31), geriatric (*n* = 15), and postacute (*n* = 10) units were asked to order the 35 interventions (from −4 the lowest to +4 the highest priority) using a Q‐sort table. Averages (confidence interval at 95%) were calculated at the group level, and a by‐person factor analysis was applied to discover underlying patterns of prioritization at the overall and at the individual levels. At the group level, “Ensuring a safe environment (e.g., reducing bed height)” was ranked as the highest priority (2.29 out of four); at the individual level, three prioritization patterns accounting for a total variance of 50.21% have emerged: “Individual needs‐oriented” (33.82% variance explained; 41 nurses); “Prevention‐oriented” (8.47%; five nurses); and “Cognitive‐oriented” (7.92%; six nurses). At the group level, nurses prioritize safety while caring for patients with delirium; however, at the individual level, they follow three different patterns of prioritization oriented toward diverse aspects, suggesting uncertainty in the actions to be taken—with potential implications for patients.

## 1 Introduction

Patients with delirium are defined as being affected by disturbances in attention (i.e., reduced ability to direct, focus, sustain, and shift attention) and awareness (reduced orientation to the environment), usually with a rapid onset and fluctuating course (American Psychiatric Association 2022). When affected by this condition, patients have a greater need for tailored nursing care to prevent adverse outcomes (e.g., falls, self‐injuries) (Al Huraizi et al. 2023; Faeder et al. 2023). However, despite the available evidence‐based interventions specifically for hospitalized patients (National Institute for Health and Care Excellence NICE 2023), patients with delirium may receive not only poor nursing care but also wrong treatments (e.g., restraints, insufficient fluid intake), thus further increasing the stimuli triggering episodes of delirium (e.g., Strömmer et al. 2020), leading to worse individual, family, and healthcare system outcomes (Al Huraizi et al. 2023; Rosgen et al. 2020).

According to the studies available, poor nursing care of these patients is associated with a lack of knowledge regarding delirium, along with poor staff attitudes (Solberg et al. 2021) and limited human resources at the unit level (Thomas, Coleman, and Terry 2021). All these factors lead nurses to give low priority to delirium management (Lim, Lim, and Ignacio 2022). Moreover, older patients may further be ranked as a lower priority because other unstable (Marková and Jarošová 2022) and younger patients are prioritized as first (Dobrowolska et al. 2019; Mansutti et al. 2020).

Nurses decide on priorities according to so‐called “priority setting,” defined as a preferred order of care interventions, resulting in delaying activities that are deemed to be less urgent and/or important (Hendry and Walker 2004). The most highly prioritized activities are patient assessment and medication administration (Cho et al. 2020), while those ranked as low priority are strictly related to fundamental needs (e.g., mobilization, care hygiene). These prioritization patterns are shaped by external factors at the group level and by internal factors at each individual nurse's level. Among the external factors, the patient's condition and the culture of the context or environment (Marková and Jarošová 2022); the perceived lack of time; the philosophies and care models adopted by the facility (Mandal, Seethalakshmi, and Rajendrababu 2020); and the influence of relatives and that of the manager and teamwork (Abdelhadi, Drach‐Zahavy, and Srulovici 2020) have all been documented as playing a role in shaping the priorities. All these factors have an impact on the whole nursing staff; furthermore, prioritization is also influenced by the education, experience, personal values, and beliefs of each individual nurse (Drach‐Zahavy and Srulovici 2019). Therefore, the decision regarding what should be delivered first or later is shaped by the group at the unit level, and by each nurse at the individual level. However, evidence available on how priorities are decided upon has been produced only in the context of delirium prevention (Sist et al. 2024a). Producing knowledge regarding the prioritization patterns at the group and at the individual levels could facilitate decisions on how to improve the quality of care in the field of delirium management, as it is still considered suboptimal (Hoch et al. 2022).

### 1.1 Aims

The aims of the study were to describe (a) how nurses prioritize interventions to manage episodes of delirium and (b) the underlying prioritization patterns at the overall group and at the individual levels.

## 2 Methods

### 2.1 Design

**Figure 1:**
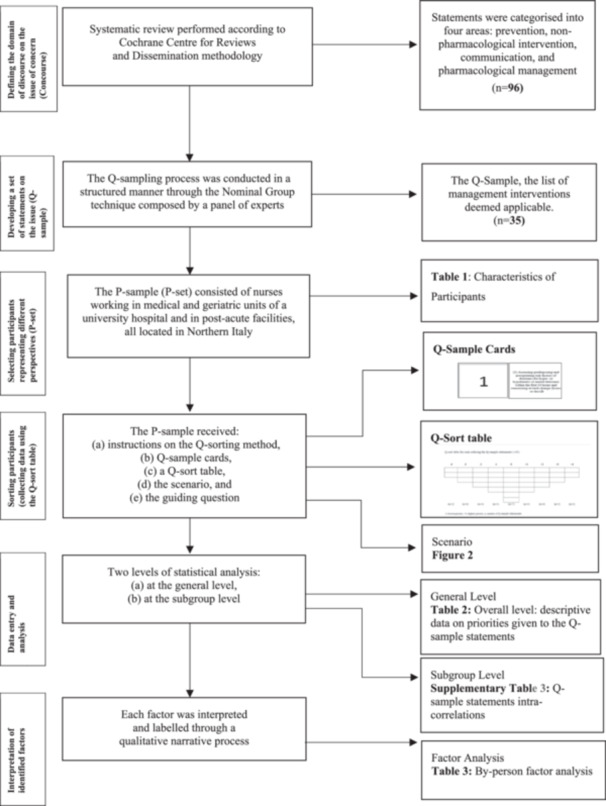
Flowchart of the Q‐methodology (Akhtar‐Danesh, Baumann, and Cordingley 2008; Simons 2013; Watts and Stenner 2005). P‐set (P‐ sample), participants; Q‐sample, statements in relation to the topic (sample Q); Q‐sort table, classification grids to allow participants to sort the statements.

In the context of a research project investigating the prioritization patterns and reasons behind them in the prevention of delirium (Sist et al. 2024a, 2024b), a Q‐methodology (Watts and Stenner 2005) was used to achieve the expected aim. The method was selected because of its capacity to discover and describe multiple points of view, starting from subjective and broadening to objective data, thus allowing the discovery and analysis of complex processes (Akhtar‐Danesh, Baumann, and Cordingley 2008; Simons 2013). The study was planned and conducted according to the available guidelines (Churruca et al. 2021) as summarized in Supporting Information S1: Table 1 and in Figure 1.

### 2.2 Defining the Domain of Discourse on the Issue of Concern (Concourse)

**Figure 2:**
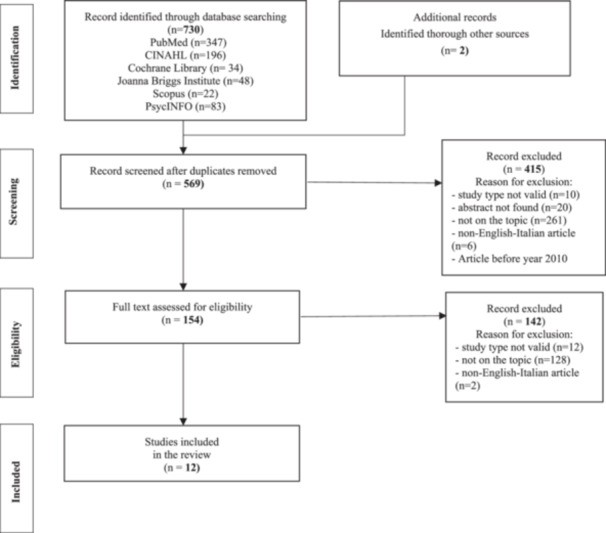
Flowchart of studies screening process (Page et al. 2021). CINAHL, Cumulative Index to Nursing and Allied Health Literature; PRISMA, Preferred Reporting Items for Systematic Reviews and Meta‐Analyses; PsycINFO, Psychological Information Database.

First, to develop the Issue of Concern (concourse) (Akhtar‐Danesh, Baumann, and Cordingley 2008) a list of evidence‐based interventions recommended for patients with delirium in medical, geriatric, and postacute settings was identified through a systematic review following the Center for Reviews and Dissemination Systematic Reviews (2009). Two researchers (N. V. U., L. S.) and a third in case of disagreements (M. P.), conducted the systematic review. By accessing the Cochrane Library, PubMed, Scopus, Cumulative Index to Nursing and Allied Health Literature, Psychological Information, and Joanna Briggs Institute databases in January 2021, specific keywords (“nursing management,” “nursing intervention”) and MesH terms (“delirium,” “delirium/nursing,” “delirium/prevention and control,” “activities of daily living,” “nursing care,” and “patient care management”) were applied. Primary and secondary studies written in English or Italian, with abstracts available, published in between 2011 and 2021, were eligible. There were included studies regarding (a) patients aged 65 and over and (b) cared for in medical, geriatric, and postacute settings according to the high prevalence and incidence of delirium documented (Fuchs et al. 2020). A total of seven quantitative studies (Avendaño‐Céspedes et al. 2016; Boockvar, Teresi, and Inouye 2016; Hasemann et al. 2016, 2018; Rosenbloom and Fick 2014; Sepúlveda et al. 2019; Solà‐Miravete et al. 2018), three systematic reviews (Oh et al. 2017; Rosenbloom and Fick 2014; Yakimicki et al. 2019), one systematic review and meta‐analysis (Thomas et al. 2014), and a clinical guideline (Siddiqi et al. 2016) were included. Then, two reviewers (N. V. U., L. S.) independently assessed included studies and summarized the relevant interventions (Figure 2). All duplicate interventions were eliminated, and 96 statements ( = the Concourse) (Sist et al. 2022) were obtained and categorized in the following areas: preventive, nonpharmacological, communications, and pharmacological interventions (Bellelli et al. 2018; National Institute for Health and Care Excellence NICE 2023).

### 2.3 Developing a Set of Statements About the Issue (Q‐Sample)

A Q‐sampling process (Brown 1996) was conducted to identify, from the list of recommended interventions that emerged, only those applicable in the medical, geriatric, and postacute care settings. For this purpose, a nominal group (NG) technique (Foth et al. 2016) was adopted involving nurses with > 5 years of experience and with a clinical, research, educational, and/or managerial background or responsibilities as suggested by the methodology (Brown 1996). After having identified the NG members and invited them to participate in a consensus meeting (L. S.), the following steps were performed (Foth et al. 2016): (a) a round‐robin, in which all participants were provided with the list of recommended interventions that emerged from the literature to prepare them to give their input at the meeting; (b) clarification of the interventions provided whereby all questions were addressed; (c) a vote on the intervention, using a four‐point Likert scale ranging from 1 (totally inapplicable) to 4 (totally applicable) in daily practice; and (d) a discussion. The meeting was conducted online, and the vote was collected using the Wooclap platform, a web‐based interactive electronic platform used to create polls, questionnaires, and quizzes.

From the agreed list of 51 interventions with a mean score of ≥ 3.5, three researchers reread the comments provided by the NG members and identified the final list of interventions by: (a) eliminating those that were not specific (*n* = 2); (b) adding two suggested; (c) collapsing some according to their similarities (*n* = 16); and (d) making explicit educational interventions for the family and/or caregiver indicating the contents and the tools. The results were subjected to a further member review (Birt et al. 2016), producing the Q‐sample of 35 interventions aimed at managing delirium episodes in elderly patients hospitalized in medical, geriatric, and postacute settings (Sist et al. 2022).

### 2.4 Selecting Participants Representing Different Perspectives (P‐Set)

The P‐sample (P‐set) was identified among clinical nurses working in medical, geriatric, and postacute units of an academic hospital in northern Italy. The units were decided according to their mission to admit older individuals with different clinical conditions and at high risk of developing delirium (Fuchs et al. 2020). The recommended P‐sample size of 20–60 participants (Zabala, Sandbrook, and Mukherjee 2018) was considered, therefore, 40 participants were invited by the nurse managers, three or four for each unit. Specifically, those nurses (a) with at least 6 months of experience in the unit (Drach‐Zahavy and Srulovici 2019; Ludlow et al. 2020), (b) working full‐time, and (c) willing to participate were included. All nurses contacted agreed to participate.

### 2.5 Sorting Participants (Collecting Data Using the Q‐Sort Table)

**Figure 3:**
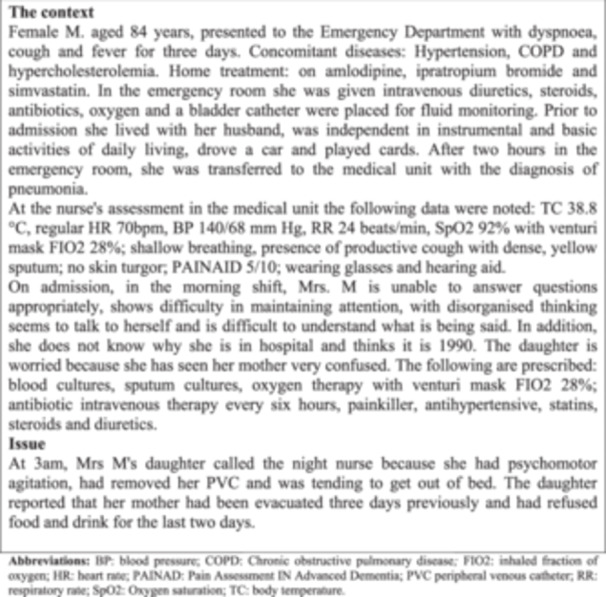
Scenario: the context and the issue.

Nurses willing to participate received the study materials from the researchers in a sealed envelope containing: information regarding the study aims and procedures, the informed consent form, and the Q‐sample cards, each showing a number randomly assigned to the recommended interventions on the front and a description of the intervention on the back. Subsequently, nurses provided their written consent to participate and sent it to the researchers by email; the link to participate in the online data collection meeting via the Zoom platform was then sent to each. The meeting was divided into four sections (Simons 2013; Watts and Stenner 2005):

a. Instructions: Participants received information regarding the Q‐sort table, with the spaces on the left representing the lowest priority (‐4) and those on the right the highest (+4) (Simons 2013) (Figure 1).

b. Scenario presentation and guiding question: The scenario was read by participants and the following question was posed by the researcher: in what order would you decide to provide the interventions to manage the episode of delirium in this patient? Please order the cards containing the interventions within the Q‐sort table from the highest (+4) to the lowest (−4) priority. Clarifications were provided regarding the scenario and the listed interventions (Figure 3).

c. Sorting the priorities: Each participant was required to order the Q‐sample statements in the Q‐sort table individually, using paper material previously provided with the following instructions (Watts and Stenner 2012):

— “Please organize the 35 Q‐sample interventions according to the scenario given into three piles: 14 at high priority, seven at neutral priority, and 14 at low priority”;
— “Please select the Q‐sample interventions from the high priority, neutral priority, and low priority stacks and reorder them in a consecutive sequence within the Q‐sort table”;
— “Please give reasons for each choice by providing notes.”

Strategies to facilitate participants were used during the meeting (e.g., rereading interventions that had not been prioritized); moreover, participants were allowed to modify the decisions during the process. During this process, researchers turned off the cameras to leave participants free to continue but remained available to answer any doubts; no interpretive suggestions were offered, but the importance of their free prioritization was emphasized (Watts and Stenner 2012).

d‐. Data collection and final interview: At the end, the participants sent to the researchers (L. S., M. P.) via WhatsApp the picture of the Q‐sort table with the ordered Q‐sample. Then, the participants were asked to fill in a short sociodemographic (e.g., age, gender) and professional (e.g., nursing education) form via the Wooclap platform.

The online meeting lasted around 2 h; it was led by one researcher (L. S.) and supported by a second (M. P.), who also took notes (e.g., interruptions) as suggested by the methodology (Watts and Stenner 2005).

### 2.6 Data Entry and Analysis

Data contained in the pictures were transferred into an Excel matrix; the analysis was performed (Akhtar‐Danesh, Baumann, and Cordingley 2008; Watts and Stenner 2005) at three levels.

a. At the overall group level, the priority given to all interventions by participants was described as a common viewpoint. Averages and 95% confidence intervals (CIs) were calculated according to the priorities assigned to each Q (−4 to +4). Moreover, with the aim of exploring correlations, if any, coefficients between Q‐sorts were calculated (Spearman's rho test) and interpreted according to Cohen's criteria as (small 0.10–0.29); medium (0.30–0.49); large (0.50–1.00) (Cohen 1988).
b. At the individual level, an inductive approach was used through the technique of exploratory factor analysis (EFA) to establish the factor (or factors) describing the underlying patterns of prioritization by using Stata 15.1 (StataCorp LLC, College Station, TX 77845, USA). The oblique rotation technique (Oblimin) was applied and the number of factors to be extracted was based on the inspection of the scree plot and the criterion of the correlation matrix > 1.
c. Differences in patterns among nurses working in different units were assessed using ANOVA F‐test with post hoc comparisons with Bonferroni corrected probability level. The statistical significance was set at *p* < 0.05.

### 2.7 Interpretation of Identified Factors

Four researchers (L. S., N. V. U., S. C. 3, A. P.) were involved in interpreting and labeling the factors (Akhtar‐Danesh, Baumann, and Cordingley 2008) by approaching the data first independently and then as a team. The researchers analyzed in depth the list of statements as grouped in the EFA; then, factors were labeled from the statements included with the highest factor loadings (Churruca et al. 2021; Watts and Stenner 2012). The labels provided by each researcher were discussed and approved by the entire research team.

### 2.8 Ethical Considerations

The research project was approved by the Bioethical Committee of the University of Bologna, Italy (Register N.0109186, 5 May 2021). All participants provided a written informed consent before entering the study.

## 3 Results

### 3.1 Population (P‐Set)

A total of 56 bedside nurses (aged 31.6 years on average, Table 1) participated; the majority were female (39; 69.6%) and educated at the university level (53; 94.6%), with some trained in the specific field of delirium (15; 26.8%). When participating in the study, they were working in medical (31; 55.4%), geriatric (15; 26.8%), and postacute units (10; 17.8%) where they gained most of their professional experience (Table 1) as shift nurses (52; 92.9%) working an average of 36.6 h/week.

**Table 1:** Characteristics of participants.

Variables	Nurses
	*N* (%)
	56 (100)
**Mean age** CI (95%)	31.6 (29.6–33.6)
**Females**	39 (69.6)
**Undergraduate education**	
Bachelor's degree in nursing	53 (94.6)
**Postgraduate education**	
Master's degree course	14 (24)
**Continuing education course(s) on delirium**	15 (26.8)
**Hospital work setting**	
Medical	31 (55.4)
Geriatrics	15 (26.8)
Postacute	10 (17.8)
**In the current unit**	
I spent the most time of my professional experience	38 (67.9)
Years of experience, mean (95% CI)	4.5 (2.7–6.2)
On‐shift	52 (92.9)
Working hours per week, mean (95% CI)	36.6 (36.1–37.2)
Overtime hours in the last 3 months, mean (95% CI)	19.8 (14.2–25.3)
**Adequacy of the nursing resources in my unit**	
100% of time	2 (3.6)
75% of time	17 (30.4)
50% of time	27 (48.2)
25% of time	8 (14.3)
0% of time	2 (3.6)
**Patients in charge in the last shift, mean** (95% CI)	16.8 (15.2–18.4)
**Newly admitted patients in the last shift, mean** (95% CI)	3.1 (2.6–3.6)
**Discharged patients in the last shift, mean** (95% CI)	2.3 (1.8–2.8)
**Satisfaction in the current role** a, **mean** (95% CI)	3.7 (3.5–3.8)
**Satisfaction with being a nurse** a, **mean** (95% CI)	4.5 (4.3–4.7)
**Satisfaction with the teamwork** a, **mean** (95% CI)	3.8 (3.5–4.0)

Abbreviation: CI, confidence interval.

^a^ From 1 (very dissatisfied) to 5 (very satisfied).

Participants felt that the number of nurses at the unit level was adequate for half of the time (27; 48.2%); moreover, they reported being responsible for an average of 16.8 patients in the last shift (3.1 newly admitted and 2.3 discharged). Regarding satisfaction, they reported an average score of 3.7 out of five (very satisfied) with the nursing role, 4.5 with being a nurse, and 3.8 with teamwork (Table 1).

### 3.2 The Prioritization Patterns at the Overall Level

At the overall level, the highest priorities identified were: “Ensuring a safe environment (e.g., reducing bed height)” (2.29; 95% CI: 1.81, 2.76); “Communicating with verbal and nonverbal language in a clear, simple way and positioning oneself in front of the person” (1.86; 95% CI: 1.40, 2.31); and “Continuous monitoring of mental (e.g., orientation, short‐ and long‐term memory) and physical state (e.g., Barthel Scale)” (1.82; 95% CI: 1.35, 2.29). On the other hand, the lowest priorities were “Providing a clock, calendar, and signs in the room (where they are and in which city)” (−2.07; 95% CI: −2.45, −1.69); and “Educating the family and/or caregiver on reorientation interventions for the person” (−1.95; 95% CI: −2.47, −1.42) (Table 2).

**Table 2:** Overall level: How nurses prioritize interventions to manage episodes delirium.

Q‐sample statements	Mean^a^	SD	95% CI
(21) Ensuring a safe environment (e.g., reducing bed height)	2.29	1.79	1.81, 2.76
(27) Communicating with verbal and nonverbal language in a clear, simple way and positioning oneself in front of the person	1.86	1.70	1.40, 2.31
(3) Continuous monitoring of mental (e.g., orientation, short‐ and long‐term memory, calculation, attention and concentration, object naming, command execution, writing, orientation in space and time, abstract reasoning, judgment) and physical state (e.g., Barthel Scale)	1.82	1.76	1.35, 2.29
(26) Communicating with the person (calling him/her by name, explaining where I am, who I am, what my role is, what activities are taking place)	1.80	1.50	1.40, 2.21
(4) Monitoring the vital parameters (heart rate, blood pressure, oxygen saturation)	1.75	2.39	1.11, 2.39
(10) Detecting issues in intestinal elimination (diarrhea and constipation)	1.52	1.64	1.08, 1.96
(17) Assessing pain with verbal and nonverbal expression or using scales (e.g., PAINAID)	1.14	1.86	0.64, 1.64
(34) Administering and monitoring the effects of administered medication (e.g., haloperidol)	1.04	1.50	0.63, 1.44
(2) Assessing the changes in the vigilance, attention, and cognitive and behavioral status within the first 24 h and demonstration of a marked change or fluctuating course in attention, comprehension, or other cognitive‐behavioral functions, reassessing at each change (hours or days) (e.g., with four AT scale)	0.93	2.10	0.37, 1.49
(1) Assessing predisposing and precipitating risk factors for delirium (for hyper‐ or hypokinetic or mixed delirium) within the first 24 h and reassessing at each change (hours or days)	0.82	1.96	0.30, 1.35
(6) Preventing restraints (physical, pharmacological, environmental, psychological, or relational restraints)	0.59	1.99	0.06, 1.12
(28) Encouraging the presence of the family and/or caregiver on a daily basis and sharing the experience of delirium with the caregiver	0.55	1.93	0.04, 1.07
(18) Minimizing the effects of the hospital environment such as noises (doorbell, alarms, pumps, monitors) and lights (avoiding direct light and using soft lights)	0.55	1.90	0.05, 1.06
(35) Treating pain (administration of medication and nonpharmacological treatments)	0.50	1.71	0.04, 0.96
(11) Detecting issues in urinary elimination (presence of bladder globus)	0.32	1.78	−0.16, 0.80
(14) Encouraging sleep by avoiding nighttime procedures	0.02	1.69	−0.43, 0.47
(8) Motivating to take oral nutrition and water according to their metabolic needs (avoiding caffeine and heavy meals in the evening)	0.00	1.68	−0.45, 0.45
(22) Minimizing the number of people in the room and placing the person in a single room (delirium room)	−0.13	1.83	−0.61, 0.36
(32) Evaluating therapy (number, dosage, pharmaceutical form of medications) together with the doctor	−0.14	2.08	−0.70, 0.42
(24) Working in teamwork, carrying out multiprofessional interventions, performing multiple interventions together	−0.14	1.64	−0.58, 0.30
(13) Assessing sleep activity and patterns	−0.21	1.33	−0.57, 0.14
(9) Encouraging the person to drink	−0.27	1.84	−0.76, 0.23
(7) Assessing the integrity, functioning, and placing of hearing, sight, and dental aids	−0.34	1.81	−0.82, 0.15
(25) Tailoring interventions according to the person's needs and the setting, trying to maintain a daily routine for the person	−0.46	1.73	−0.93, 0.00
(33) Controlling and managing medication interactions	−0.41	1.84	−0.90, 0.08
(5) Preventing infection (assessment, testing, medication administration)	−0.84	1.58	−1.26, −0.42
(12) Removing urinary catheter as soon as conditions permit and/or avoiding urinary catheterization to encourage spontaneous urination	−1.07	1.46	−1.46, −0.68
(23) Minimizing room and ward changes	−1.30	1.65	−4.0, 2.0
(31) Facilitating communications with family members and/or caregivers by phone or video call	−1.36	1.80	−1.84, ‐0.88
(15) Encouraging the person to walk and providing walking aids (appropriate and accessible)	−−1.38	1.78	−1.85, −0.90
(16) Getting the person out of bed every day	−1.71	1.36	−2.08, −1.35
(20) Encouraging the presence of personal items (photos, bedspreads)	−1.79	1.72	−2.25, −1.32
(29) Educating the family and/or caregiver. Contents: risk factors and signs and symptoms of delirium, and changes in the person. Tools: information leaflets	−1.95	1.90	−2.46, −1.44
(30) Educating the family and/or caregivers. Contents: re‐orientation interventions for the person. Tools: information leaflets	−1.95	1.97	−2.47, −1.42
(19) Providing a clock, calendar, and signs in the room (where they are and in which city)	−2.07	1.42	−2.45, −1.69

Abbreviations: CI, confidence interval; four AT, assessment test for delirium and cognitive impairment; PAINAD, Pain Assessment IN Advanced Dementia; SD, standard deviation.

^a^ From +4 as the highest priority to −4 as the lowest priority.

Four interventions were ranked on average, above 1 as a priority (with 4 as the highest priority) while nine were ranked below 1 (with −4 as the lowest priority). Additionally, while in some interventions, the priorities given were clearly different (e.g., 2.29 and 1.86 out of four for “Ensuring a safe environment” and “Communicating with verbal and nonverbal language in a clear, simple way,” respectively), in others, differences were limited or absent (0.05 and 0.14 out of four for “Motivating to take oral nutrition and water according to their metabolic needs” and “Detecting issues in intestinal elimination,” respectively).

Correlations in the priorities ranged from rho = −0.266 (*p *< 0.05) to rho = 0.802 (*p *< 0.01) (Supporting Information S1: Table 2). The strongest correlations were between the following interventions:

▪ “Educating the family and/or caregiver about risk factors, signs, and symptoms of delirium and changes in the person” and “Educating the family and/or caregiver about the reorientation interventions” (rho = 0.802, *p* < 0.01).
▪ “Assessing pain with verbal and nonverbal expression or using scales” and “Treating pain” (rho = 0.669, *p* < 0.01).

No strong negative correlations emerged (rho = < −0.500); the highest were between “Preventing infections” and “Minimizing the effects of the hospital environment, such as noise” (rho = −0.455, *p* < 0.01) (Supporting Information S1: Table 2).

### 3.3 The Prioritization Patterns at the Individual Level

Three prioritization patterns accounting for a total variance of 50.21% have emerged. Specifically, the first factor was labeled “Individual needs‐oriented” (explained variance = 33.82%), reflecting the prioritization patterns of 41 nurses; the second was labeled “Prevention‐oriented” (explained variance = 8.47%) and expressed the pattern of five nurses; and the third was labeled “Cognitive‐oriented” (=7.92%) and concerned six nurses (Table 3). Four nurses did not report a common view regarding prioritization.

**Table 3:** By‐person factor analysis: The prioritization patterns according to the nurses' individual characteristics.

Q‐sample statements	Factor 1 individual needs oriented	Factor 2 prevention oriented	Factor 3 cognitive oriented
(1) Assessing predisposing and precipitating risk factors for delirium (for hyper‐ or hypokinetic or mixed delirium) within the first 24 h and reassessing at each change (hours or days)	0	4	3
(2) Assessing the changes in the vigilance, attention, cognitive, and behavioral status within the first 24 h and demonstration of a marked change or fluctuating course in attention, comprehension, or other cognitive‐behavioral functions, reassessing at each change (hours or days) (e.g., with four AT scale)	1	3	3
(3) Continuous monitoring of mental (e.g., orientation, short‐ and long‐term memory, calculation, attention and concentration, object naming, command execution, writing, orientation in space and time, abstract reasoning, judgment) and physical state (e.g., Barthel Scale)	3	2	−1
(4) Monitoring the vital parameters (heart rate, blood pressure, oxygen saturation)	3	4	−3
(5) Preventing infection (assessment, testing, medication administration)	0	−3	0
(6) Preventing restraints (physical, pharmacological, environmental, psychological, or relational restraints)	−1	3	−1
(7) Assessing the integrity, functioning, and placing of hearing, sight, and dental aids	−1	1	−2
(8) Motivating to take oral nutrition and water according to their metabolic needs (avoiding caffeine and heavy meals in the evening)	2	3	0
(9) Encouraging the person to drink	0	1	−1
(10) Detecting issues in intestinal elimination (diarrhea and constipation)	−2	−1	−2
(11) Detecting issues in urinary elimination (presence of bladder globus)	0	−1	0
(12) Removing urinary catheter as soon as conditions permit and/or avoiding urinary catheterization to encourage spontaneous urination	0	−2	0
(13) Assessing sleep activity and patterns	−2	0	−4
(14) Encouraging sleep by avoiding nighttime procedures	−3	0	−3
(15) Encouraging the person to walk and providing walking aids (appropriate and accessible)	2	1	−2
(16) Getting the person out of bed every day	1	−2	1
(17) Assessing pain with verbal and nonverbal expression or using scales (e.g., PAINAID)	−3	−4	−1
(18) Minimizing the effects of the hospital environment such as noises (doorbell, alarms, pumps, monitors) and lights (avoiding direct light and using soft lights)	−3	−2	0
(19) Providing a clock, calendar, and signs in the room (where they are and in which city)	4	0	4
(20) Encouraging the presence of personal items (photos, bedspreads)	0	−1	−1
(21) Ensuring a safe environment (e.g., reducing bed height)	−2	−1	−1
(22) Minimizing the number of people in the room and placing the person in a single room (delirium room)	0	0	0
(23) Minimizing room and ward changes	−1	0	2
(24) Working in teamwork, carrying out multiprofessional interventions, performing multiple interventions together	3	0	1
(25) Tailoring interventions according to the person's needs and the setting, trying to maintain a daily routine for the person	4	−3	2
(26) Communicating with the person (calling him/her by name, explaining where I am, who I am, what my role is, what activities are taking place)	1	−3	4
(27) Communicating with verbal and nonverbal language in a clear, simple way and positioning oneself in front of the person	−4	−1	3
(28) Encouraging the presence of the family and/or caregiver on a daily basis and sharing the experience of delirium with the caregiver	−4	−2	2
(29) Educating the family and/or caregiver. Contents: risk factors and signs and symptoms of delirium, and changes in the person. Tools: information leaflets	−2	−4	1
(30) Educating the family and/or caregivers. Contents: re‐orientation interventions for the person. Tools: information leaflets	1	0	−4
(31) Facilitating communications with family members and/or caregivers by phone or video call	−1	2	−1
(32) Evaluating therapy (number, dosage, pharmaceutical form of medications) together with the doctor	2	2	0
(33) Controlling and managing medication interactions	2	1	−2
(34) Administering and monitoring the effects of administered medication (e.g., haloperidol)	−1	1	−3
(35) Treating pain (administration of medication and nonpharmacological treatments)	1	2	1
**Number of loading (=nurses with similar profile)**	**41**	**5**	**6**
Eigenvalues	18.94	4.74	4.43
**% of explained variance**	**33.82**	**8.47**	**7.92**

Abbreviations: four AT, assessment test for delirium and cognitive impairment; PAINAD, Pain Assessment IN Advanced Dementia.

### 3.4 Differences Across Units

There were no significant differences in the by‐person factor analysis across settings (medical, geriatric, and postacute) for the first (*p* = 0.20) and third factor (*p* = 0.51), whereas for the second, a significant difference emerged between the geriatric and medical units (ANOVA = 3.79 with *p* = 0.03) (Bonferroni difference between the mean values 0.81; *p* = 0.025).

## 4 Discussion

We considered the most applicable evidence‐based interventions according to bedside nurses (Sist et al. 2024a) and a scenario describing a patient with delirium. The aim was to describe priority patterns at the overall level and to capture the subjectivity of nurses as individuals to explore subgroup patterns (Akhtar‐Danesh, Baumann, and Cordingley 2008) in medical, geriatric, and postacute settings where a high occurrence of delirium has been documented (Fuchs et al. 2020). Overall, the study involved full‐time nurses, mostly satisfied with their role, caring for a high number of patients with a perceived lack of resources, reflecting the main profile of Italian nurses in line with previous studies (Palese et al. 2020).

Findings suggest that the prioritization process may be seen from two perspectives: the first as undertaken by nurses as a whole, and the second according to individual nurses. These two perspectives demonstrated different patterns; thus, there was variability in setting priorities while caring for patients with delirium. The etiology behind delirium can be multiple and interactive in nature, thus a pattern of uncertainty in the actions to be undertaken in patient management is understandable; however, different priorities identified by nurses may be informed by knowledge and attitudes suggesting efforts in disseminating the best recommendations (Devlin et al. 2018; National Institute for Health and Care Excellence NICE 2023) and in harmonizing their decisions given that uncertainty in delirium management may fragment the care and lead to negative impacts on patients.

### 4.1 The Prioritization Patterns at the Overall Level

Nurses prioritize the safety of the environment and then communication with the patient; after these interventions, nurses prioritize monitorization of cognitive and physical status, and vital signs, bowel elimination, and pain assessment. The differences in the averages between the first and the second priority (ensuring a safe environment vs. communication) indicate a clear prioritization of safety. This may be influenced by the delirium safety culture (Kim and Moon 2023) that has been emphasized in recent years to avoid adverse events such as falls or self‐harm (Faeder et al. 2023), leading to medico‐legal consequences (Eost‐Telling et al. 2024); however, excessive attention to safety may limit the freedom of patients up to the administration of sedatives to calm them and prevent self‐injuries (Veronese et al. 2024).

As a second priority, nurses indicated communication, which may reflect their attempt to reassure the patients, suggesting a different pattern to that documented where emotional and communication support is the care most widely missed among hospitalized patients (Mainz et al. 2024). Then, nurses prioritize interventions aimed at monitoring (vital signs, pain), with no clear differences between them according to their averages: Nurses seem to take into consideration the multiple and interactive etiology of delirium (e.g., pain, constipation; Devlin et al. 2018; National Institute for Health and Care Excellence NICE 2023) with a tendency to monitor them rather than act to detect the underlying causes of delirium (Shrestha and Fick 2020). In contrast, interventions recommended by guidelines (Devlin et al. 2018; National Institute for Health and Care Excellence NICE 2023) emphasizing the need to provide a personalized environment (e.g., “Provide a clock, calendar, and signs in the room,” “Minimize room and ward changes”), as well as caregiver and family education, have been less prioritized. Findings may reflect the pandemic period in which the study was conducted; however, they suggest the need to reconsider the role of the personalized environment and that of family members as a reference point for patients with delirium (Lee et al. 2023).

Regarding the correlations, although they do not express causality, their squared coefficients may indicate the amount of variability in one intervention that has been shared with another. Specifically, family/caregiver education regarding risks and reorientation interventions (rho = 0.802, *R* ^2^ = 0.895) are closely connected, suggesting that nurses give the same priority and thus perform these interventions close to each other at the same time. Similarly, the strong correlation between “Assessing pain” and “Treating pain” (rho = 0.669, *R* ^2 ^= 0.817) may reflect the logical consequence in the clinical reasoning (assessing and then treating) and the priority given to pain and its treatment in several clinical settings as recommended (Devlin et al. 2018; National Institute for Health and Care Excellence NICE 2023). Overall, most correlations that emerged are positive and statistically significant, suggesting that nurses may have viewed the interventions as bundles or packages, with the aim of applying them simultaneously to maximize their capacity and resources and to increase effectiveness (Zhao et al. 2023). In contrast, only one strong negative correlation emerged (“Preventing infections” and “Minimizing the effects of the hospital environment such as noise”) (rho = −0.455; *R* ^2 ^= 0.674), suggesting that these interventions are applied at different times. Overall, only one intervention was not correlated with the others, namely “Tailoring interventions according to the person's needs and the setting, trying to maintain a daily routine for the person”; this may reflect the general approach toward these patients, as recommended (Devlin et al. 2018; National Institute for Health and Care Excellence NICE 2023).

### 4.2 The Prioritization Patterns at the Individual Level Across Units

The by‐person factor analysis reveals three profiles, suggesting the existence of three prioritization patterns: “Individual needs‐oriented” with 41 nurses; “Prevention‐oriented” with five nurses; and “Cognitive‐oriented” with six nurses. The remaining four nurses did not report a common view regarding the prioritization process.

Although each pattern has been characterized by a fragmented flow of priorities, with some difficulties in labeling each, the first is shaped around the individualization of care whereby actions are tailored according to needs. Nurses shape their interventions according to each patient's needs. On the one hand, this may be considered the maximum expression of individualized nurses' care; on the other, they may be overwhelmed with too many patients assigned and thus find it difficult to identify needs, resulting in lack of care (Cura Della Redazione 2024) and discontinuity across shifts.

The second pattern (“Preventive‐oriented”) suggests that when delirium episodes occur, as in the case reported in the scenario, nurses still prioritize prevention, with several interventions. Thus, nurses seem to focus on the need to prevent further factors that may prolong delirium episodes; however, they seem to wait to take the time to take actions likelihood to better understand how delirium develops to decide which actions to take, all resulting in the risk of postponed effective treatments of delirium (Cengia et al. 2024; Francis and Young 2023).

The third pattern has been labeled “Cognitive‐oriented” because it implies communication and cognitive reorientation. In this context, communication is ranked as important, as also suggested by the literature (Devlin et al. 2018; National Institute for Health and Care Excellence NICE 2023).

While for the first and the third pattern no differences across settings emerged, suggesting similarities in the priorities, the second pattern (“Preventive‐oriented”) reported significant differences, implying that geriatric units are more focused on delirium prevention than medical units are, reflecting their attitude to implement patient‐centered management models with a multiprofessional approach.

Overall, the patterns that emerged seem to delineate three different ways to manage delirium in which four nurses are not included, thus suggesting the existence of additional individual patterns of prioritization that should be discovered (Drach‐Zahavy and Srulovici 2019). Furthermore, only half of the explained variance has emerged, suggesting that more research is needed. On the one side, the existence of diverse patterns of prioritization across nurses may be seen as expression of the richness of the clinical reasoning of nurses; on the other, these may introduce inconsistencies in daily practice where the patient with delirium is expected to be cared for with an evidence‐based set of interventions; moreover, these inconsistencies may affect the quality of the care, nurses' satisfaction, and the satisfaction with the care received by patients and families.

### 4.3 Limitations

This study has several limitations. The first relates to the Q‐sample (the list of management interventions), which was drawn from the literature and then checked for applicability by consensus (Sist et al. 2022). In the systematic review, we included both primary and secondary studies (systematic reviews) without reading the articles included in the systematic reviews. The selection of the interventions may have resulted in a kind of over‐prioritization reducing the possible interventions to those mostly investigated in the available literature. In the future, a more inclusive approach to the literature search is suggested, considering primary studies also in the case of systematic reviews and extending the timeframe. A more systematic approach may help in updating the review and in following‐up changes in the patterns of priorities. Furthermore, it is suggested to leave participants free to identify the interventions to better discover the underlying criteria influencing the prioritization without any external imposition as applied in our study where only recommended interventions have been ordered in their priority. Second, a scenario was used to trigger the reasoning, and despite the attempts to provide a realistic situation, the limited description offered to prevent distractions may have influenced the priorities identified by participants (Abdelhadi, Drach‐Zahavy, and Srulovici 2020). Third, the data collection was performed online, and this may have also prevented an in‐depth engagement in the process (Simons 2013). Additionally, the study only involved nurses, yet delirium management is a multidisciplinary approach, and its treatment is an interdependent function of nurses, depending on investigation and elimination of various clinical hypotheses. Moreover, participant nurses were not assessed in terms of their knowledge, competence, attitudes, and experiences regarding delirium management. Furthermore, data were collected during the pandemic when the circumstances experienced by the nurses may have influenced their priorities, so accumulating evidence through postpandemic studies is strongly recommended.

## 5 Conclusions

To the best of our knowledge, this is the first study involving a Q‐methodology to detect how nurses prioritize interventions aimed at caring for patients with delirium in acute and postacute hospital settings. At the group level, nurses attribute high priority to interventions aimed at ensuring safety, followed by those ensuring communication and continuing surveillance by monitoring patients' conditions. They also attribute low priority to family involvement and to changing the features of the environment to ensure a calm setting. At the individual level, three different patterns of prioritization emerged: oriented to individual needs, to prevention, and to cognition. On the one hand, different patterns may reflect the attempt to tailor nursing care according to the situation as perceived by nurses; however, diverse patterns of prioritization may introduce fragmentation in the care, diverse plans for action across shifts, and an unclear care pathway. Ultimately, they may affect the quality of care and cause variations across nurses and shifts, introducing additional issues to cope with when dealing with patients experiencing delirium. Future studies are suggested to understand priority patterns in a multidisciplinary delirium treatment approach and the potential effects of different patterns at the patient, family, and nurse levels.

## Author Contributions

**Luisa Sist:** conceptualization, methodology, data curation, formal analysis, writing–original original draft, writing–review and editing. **Matteo Pezzolati, Nikita Valentina Ugenti, Silvia Cedioli, Stefania Chiappinotto:** supervision, writing–review and editing. **Paola Rucci:** conceptualization, methodology, writing–review and editing, data curation. **Rossella Messina:** writing–review and editing. **Alvisa Palese:** conceptualization, methodology, project administration, writing–original draft, writing–review and editing. All authors read and approved the final manuscript. Each author participated sufficiently in the work to take public responsibility for appropriate portions of the content. **Luisa Sist, Matteo Pezzolati, Nikita Valentina Ugenti, Silvia Cedioli, Stefania Chiappinotto, Rossella Messina, Paola Rucci**, and **Alvisa Palese:** agreed to be accountable for all aspects of the work in ensuring that questions related to the accuracy or integrity of any part of the work are appropriately investigated and resolved.

## Conflicts of Interest

The authors declare no conflicts of interest.

## Supporting information

Supporting information.

## Data Availability

The data that supports the findings of this study are available in the supplementary material of this article.
