# Supplementary material for: Prioritization Patterns of Nurses in the Management of a Patient With Delirium: Results of a Q‐Methodology Study
Source: Res Nurs Health. 2025 Feb 2;48(2):257–70. doi: 10.1002/nur.22449 (PMC11873757; doi:10.1002/nur.22449)
Supplement: Supplementary file 1 — Supporting information. [file NUR-48-257-s001.docx]

# **SUPPLEMENTARY TABLE 1: Checklist of information to include when reporting a Q-methodological study (**Churruca et al., 2021)

| **Items** | **Section(s)** |
| --- | --- |
| How the statements were refined and reduced to produce the draft and final Q-set | See Defining the domain of discourse on the issue of concern (Concourse) |
| The number of statements in the final Q-set | See Defining the domain of discourse on the issue of concern (Concourse) |
| What, if any, piloting was done and what the results were | Not performed |
| The materials used for the Q-sorting task including the ranking scale and anchors | See Sorting participants (collecting data using the Q-sort table) |
| How the Q-sorting task was administered | See Sorting participants (collecting data using the Q-sort table) |
| What, if any, other methods were used in conjunction with Q-sorting, and how the data captured by these methods was used in relation to Q-data | Not appropriate |
| The techniques used for factor extraction and rotation | See Data Entry and Analysis |
| The software programs used to administer and/or analyse the data | See Data Entry and Analysis |
| The information used to decide the number of factors to extract, rotate and interpret | See Data Entry and Analysis |
| The amount of variance explained by the factor solution | See The prioritisation patterns at the individual level |
| The processes for interpreting the factors | See Interpretation of identified factors |
| A rich narrative for each factor that explains the shared meaning it represents, supported by Q-set statements, and participant quotes where available | See The prioritisation patterns at the individual level; see Table 3 |

|  |
| --- |
|  |
|  |
|  |
|  |
|  |
|  |
|  |
|  |
|  |
|  |
|  |
|  |

**SUPPLEMENTARY TABLE 2 Q-sample statements intra-correlations: findings**

| **Q-sample statement(s)**  **Reference statement** | **Q-sample statement(s)** | **Rho** | **Q-sample statement(s)** | **Rho** |
| --- | --- | --- | --- | --- |
| (1) Assessing predisposing and precipitating risk factors of delirium (for hyper- or hypokinetic or mixed delirium) within the first 24 hours and reassessing at each change (hours or days) | (2) Assessing the changes in the vigilance, attention, cognitive and behavioural status within the first 24 hours and demonstration of a marked change or fluctuating course in attention, comprehension or other cognitive-behavioural functions; Reassessing at each change (hours or days) (e.g. with 4 AT scale) | 0.419** | (21) Ensuring a safe environment (e.g reducing bed height) | -0.313* |
|  | _ | _ | (26) Communicating with the person (calling him/her by name, explaining where I am, who I am, what my role is, what activities are taking place) | -0.306* |
|  | _ | _ | (7) Assessing the integrity, functioning and placing hearing, sight and dental aids | -0.301* |
|  | _ | _ | (13) Assessing sleep activity and patterns | -0.287* |
| (2) Assessing the changes in the vigilance, attention, cognitive, and behavioural status within the first 24 hours and demonstration of a marked change or fluctuating course in attention, comprehension, or other cognitive-behavioural functions, reassessing at each change (hours or days) (e.g., with 4 AT scale) | _ | _ | (32) Evaluating therapy (number, dosage, pharmaceutical form of medications) together with the doctor | -0.363* |
|  | _ | _ | (7) Assessing the integrity, functioning and placing hearing, sight and dental aids | -0.295* |
| (3) Continuous monitoring of mental (e.g., orientation, short- and long-term memory, calculation, attention and concentration, object naming, command execution, writing, orientation in space and time, abstract reasoning, judgement) and physical state (e.g., Barthel Scale) | (33) Controlling and managing medication interactions | 0.335* | (11) Detecting issues in urinary elimination (presence of bladder globus) | -0.313* |
| (4) Monitoring the vital parameters (heart rate, blood pressure, oxygen saturation) | (35) Treating pain (administration of medication and non-pharmacological treatments) | 0.293* | (14) Encouraging sleep by avoiding nighttime procedures | -0.316* |
|  | (5) Preventing infection (assessment, testing, medication administration) | 0.270* | (21) Ensuring a safe environment (e.g., reducing bed height) | -0.293* |
|  | _ | _ | (22) Minimising the number of people in the room and placing the person in a single room (delirium room) | -0.288* |
|  | _ | _ | (23) Minimising room and ward changes | -0.283* |
|  | _ | _ | (28) Encouraging the presence of the family and/or caregiver daily and sharing the experience of delirium with the caregiver | -0.277* |

| **Q-sample statement(s)**  **Reference statement** | **Q-sample statement(s)** | **Rho** | **Q-sample statement(s)** | **Rho** |
| --- | --- | --- | --- | --- |
| (5) Preventing infection (assessment, testing, medication administration) | (12) Removing urinary catheter as soon as conditions permit and/or avoiding urinary catheterisation to encourage spontaneous urination | 0.269* | (18) Minimising the effects of the hospital environment such as noises (doorbell, alarms, pumps, monitors) and lights (avoiding direct light and using soft lights) | -0.455** |
|  | _ | _ | (14) Encouraging sleep by avoiding nighttime procedures | -0.405** |
|  | _ | _ | (28) Encouraging the presence of the family and/or caregiver on a daily basis and sharing the experience of delirium with the caregiver | -0.306* |
|  | _ | _ | (13) Assessing sleep activity and patterns | -0.277* |
| (6) Preventing restraints (physical, pharmacological, environmental, psychological, or relational restraints) | (22) Minimising the number of people in the room and placing the person in a single room (delirium room) | 0.272* | (8) Motivating to take oral nutrition and water according to their metabolic needs (avoiding caffeine and heavy meals in the evening) | -0.270* |
|  | (23) Minimising room and ward changes | 0.266* | _ | _ |
| (7) Assessing the integrity, functioning, and placing of hearing, sight, and dental aids | (27) Communicating with verbal and non-verbal language in a clear, simple way and positioning oneself in front of the person |  | (34) Administering and monitoring the effects of administered medication (e.g., haloperidol) | -0.382** |
|  | _ | _ | (33) Controlling and managing medication interactions | -0.304* |
| (8) Motivating to take oral nutrition and water according to their metabolic needs (avoiding caffeine and heavy meals in the evening) | (9) Encouraging the person to drink | 0.408** | (22) Minimising the number of people in the room and placing the person in a single room (delirium room) | -0.400** |
|  | (16) Getting the person out of bed every day | 0.336* | (21) Ensuring a safe environment (e.g., reducing bed height) | -0.379** |
|  | (10) Detecting issues in intestinal elimination (diarrhoea and constipation) | 0.304* | (28) Encouraging the presence of the family and/or caregiver on a daily basis and sharing the experience of delirium with the caregiver | -0.351* |
|  | _ | _ | (19) Providing a clock, calendar, and signs in the room (where they are and in which city) | -0.333* |
| (9) Encouraging the person to drink | (16) Getting the person out of bed every day | 0.275* |  |  |
| (10) Detecting issues in intestinal elimination (diarrhoea and constipation) | (35) Treating pain (administration of medication and non-pharmacological treatments) | 0.279* | (21) Ensuring a safe environment (e.g., reducing bed height) | -0.270* |
|  | (11) Detecting issues in urinary elimination (presence of bladder globus) | 0.264* | _ | _ |
|  | (15) Encouraging the person to walk and providing walking aids (appropriate and accessible) | 0.264* | _ | _ |
| (11) Detecting issues in urinary elimination (presence of bladder globus) | (12) Removing urinary catheter as soon as conditions permit and/or avoiding urinary catheterisation to encourage spontaneous urination | 0.325* | (31) Facilitating communications with family members and/or caregivers by phone or video call | -0.295* |
|  | (35) Treating pain (administration of medication and non-pharmacological treatments) | 0.304* | (18) Minimising the effects of the hospital environment such as noises (doorbell, alarms, pumps, monitors) and lights (avoiding direct light and using soft lights) | -0.272* |
|  | _ | _ | (22) Minimising the number of people in the room and placing the person in a single room (delirium room) | -0.267* |

| **Q-sample statement(s)**  **Reference statement** | **Q-sample statement(s)** | **Rho** | **Q-sample statement(s)** | **Rho** |
| --- | --- | --- | --- | --- |
| (12) Removing urinary catheter as soon as conditions permit and/or avoiding urinary catheterisation to encourage spontaneous urination | (15) Encouraging the person to walk and providing walking aids (appropriate and accessible) | 0.342** | (30) Educating the family and/or caregivers. Contents: re-orientation interventions for the person. Tools: information leaflets | -0.349** |
|  | _ | _ | (14) Encouraging sleep by avoiding nighttime procedures | -0.340* |
| (13) Assessing sleep activity and patterns | (14) Encouraging sleep by avoiding nighttime procedures | 0.473** | _ | _ |
| (14) Encouraging sleep by avoiding nighttime procedures | (18) Minimising the effects of the hospital environment such as noises (doorbell, alarms, pumps, monitors) and lights (avoiding direct light and using soft lights) | 0.406** | _ | _ |
|  | (23) Minimising room and ward changes | 0.286* | _ | _ |
| (15) Encouraging the person to walk and providing walking aids (appropriate and accessible) | (16) Getting the person out of bed every day | 0.294* | (19) Providing a clock, calendar, and signs in the room (where they are and in which city) | -0.276* |
| (16) Getting the person out of bed every day | _ | _ | (28) Encouraging the presence of the family and/or caregiver on a daily basis  and sharing the experience of delirium with the caregiver | -0.397** |
|  | _ | _ | (19) Providing a clock, calendar, and signs in the room (where they are and in which city) | -0.294* |
|  | _ | _ | (30) Educating the family and/or caregivers. Contents: re-orientation interventions for the person. Tools: information leaflets | -0.268* |
| (17) Assessing pain with verbal and non-verbal expression or using scales (e.g., PAINAID) | (35) Treating pain (administration of medication and non-pharmacological treatments) | 0.669** | (22) Minimising the number of people in the room and placing the person in a single room (delirium room) | -0.403** |
|  | (32) Evaluating therapy (number, dosage, pharmaceutical form of medications) together with the doctor | 0.268* | _ | _ |
| (18) Minimising the effects of the hospital environment such as noises (doorbell, alarms, pumps, monitors) and lights (avoiding direct light and using soft lights) | (22) Minimising the number of people in the room and placing the person in a single room (delirium room) | 0.364** | (24) Working in teamwork, carrying out multi-professional interventions, performing multiple interventions together | -0.338* |
|  | _ | _ | (33) Controlling and managing medication interactions | -0.295* |
| (19) Providing a clock, calendar, and signs in the room (where they are and in which city) | (20) Encouraging the presence of personal items (photos, bedspreads) | 0.416** | _ | _ |
|  | (27) Communicating with verbal and non-verbal language in a clear, simple way and positioning oneself in front of the person | 0.346** | _ | _ |
|  | (26) Communicating with the person (calling him/her by name, explaining where I am, who I am, what my role is, what activities are taking place) | 0.272* | _ | _ |
| (21) Ensuring a safe environment (e.g., reducing bed height) | (28) Encouraging the presence of the family and/or caregiver on a daily basis  and sharing the experience of delirium with the caregiver | 0.354** | (33) Controlling and managing medication interactions | -0.290* |

| **Q-sample statement(s)**  **Reference statement** | **Q-sample statement(s)** | **Rho** | **Q-sample statement(s)** | **Rho** |
| --- | --- | --- | --- | --- |
| (22) Minimising the number of people in the room and placing the person in a single room (delirium room) | (23) Minimising room and ward changes | 0.386** | (35) Treating pain (administration of medication and non-pharmacological treatments) | -0.281* |
|  | _ | _ | (24) Working in teamwork, carrying out multi-professional interventions, performing multiple interventions together | -0.266* |
| (23) Minimising room and ward changes | _ | _ | (29) Educating the family and/or caregiver. Contents: risk factors and signs and symptoms of delirium, and changes in the person. Tools: information leaflets | -0.305* |
| (26) Communicating with the person (calling him/her by name, explaining where I am, who I am, what my role is, what activities are taking place) | (27) Communicating with verbal and non-verbal language in a clear, simple way and positioning oneself in front of the person | 0.411** | (33) Controlling and managing medication interactions | -0.306* |
| (27) Communicating with verbal and non-verbal language in a clear, simple way and positioning oneself in front of the person | _ | _ | (34) Administering and monitoring the effects of administered medication (e.g., haloperidol) | -0.314* |
|  | _ | _ | (29) Educating the family and/or caregiver. Contents: risk factors and signs and symptoms of delirium, and changes in the person. Tools: information leaflets | -0.281* |
|  | _ | _ | (33) Controlling and managing medication interactions | -0.267* |
| (29) Educating the family and/or caregiver. Contents: risk factors and signs and symptoms of delirium, and changes in the person. Tools: information leaflets | (30) Educating the family and/or caregivers. Contents: re-orientation interventions for the person. Tools: information leaflets | 0.802** | _ | _ |
| (33) Controlling and managing medication interactions | (34) Administering and monitoring the effects of administered medication (e.g., haloperidol) | 0.454** | _ | _ |

* p = < 0.05; **p = < 0.01.

^#^ No significant correlations emerged for the following Q-sample statements: (25) Tailoring interventions according to the person's needs and the setting, trying to maintain a daily routine for the person

**Legend**: PAINAD: Pain Assessment IN Advanced Dementia; 4AT: Assessment test for delirium & cognitive impairment, Rho: correlation coefficient; Cohen's criteria (small rho = 0.10 to 0.29; medium Rho = 0.30 to 0.49; large Rho = 0.50 to 1.00) (Cohen, 1988)
